# Editorial: Research Efforts, Challenges, and Opportunities in Mitigating Aflatoxins in Food and Agricultural Crops and Its Global Health Impacts

**DOI:** 10.3389/fmicb.2022.881858

**Published:** 2022-03-22

**Authors:** Mehdi Razzaghi-Abyaneh, Zhi-Yuan Chen, Masoomeh Shams-Ghahfarokhi, Mahendra Rai

**Affiliations:** ^1^Department of Mycology, Pasteur Institute of Iran, Tehran, Iran; ^2^Department of Plant Pathology and Crop Physiology, Louisiana State University Agricultural Center, Baton Rouge, LA, United States; ^3^Department of Mycology, Faculty of Medical Sciences, Tarbiat Modares University, Tehran, Iran; ^4^Department of Biotechnology, Sant Gadge Baba Amravati University, Amravati, India; ^5^Department of Microbiology, Nicolaus Copernicus University, Torun, Poland

**Keywords:** aflatoxins, *Aspergillus flavus*, agriculture, public health, biocontrol, *Zea mays*, transgenic plants, gene silencing

Aflatoxins are a group of polyketide mycotoxins that are produced during fungal development as secondary metabolites mainly by members of the *Aspergillus* section *Flavi* (Yu et al., 2004; Norlia et al., 2019; Uka et al., 2019). Contamination of food, feed and agricultural commodities by aflatoxins impose an enormous economic concern, as these chemicals are highly carcinogenic, they can directly influence the structure of DNA (Bbosa et al., 2013; Feng et al., 2016). They can lead to fetal maldevelopment and miscarriages, and are known to suppress immune systems (Ahmed Adam et al., 2017). In a global context, aflatoxin contamination is considered a perennial concern between the 35N and 35S latitude where developing countries are mainly situated. With the expansion of these boundaries, aflatoxins are increasingly becoming a problem in countries that previously did not have to worry about aflatoxin contamination. Given the continuing problems arising from aflatoxin contamination of food and agricultural commodities throughout the world, aflatoxins research is becoming one of the most exciting and rapidly developing areas of microbial toxins research. The applications include many disciplines, from medicine to agriculture. Nowadays, traditional research on aflatoxins has been expanded to modern technologies such as omics for understanding the regulation of aflatoxin biosynthetic pathway genes, the taxonomy, ecology, biochemistry, and evolution of aflatoxigenic fungi in addition to strategies to pre- and post-harvest management of aflatoxin contamination. This includes improving host resistance of susceptible crops such as cotton, maize, peanut, and tree nuts via genetic engineering.

The present Research Topic includes one review article, one mini-review and fifteen original research articles. Contributors highlighted challenges and opportunities in mitigating aflatoxins in food and agricultural crops and the current knowledge on the global health issues of aflatoxins and aflatoxigenic fungi. All aspects of aflatoxin contamination of food and agricultural crops from epidemiology to ecology, biochemistry, molecular biology, biocontrol strategies, natural inhibitors of fungal growth and aflatoxin production, transgenic hosts and pre- and post-harvest management strategies have been discussed.

Host resistance is an attractive area of aflatoxin research pertaining to various aspects of *A. flavus*-plant host interaction. In this context, the role of signaling pathway genes, gene silencing, and development of maize inbred in association with aflatoxin reduction *in vitro* and in field conditions have been extensively studied by different researchers. Parish et al. conducted a genome-wide survey of maize genes involved in maize-fungus interaction and signaling pathways by investigating the gene expression levels among the 12 maize QTL-NILs (quantitative trait loci-near isogenic lines) that carry maize resistance QTL regions. Seven calcium-dependent protein kinases and one respiratory burst oxidase displayed significant differential expression levels among the maize QTL-NILs. The authors concluded that the elucidation of differentially expressed signaling pathway genes involved in maize resistance to *A. flavus* can provide insights into maize disease resistance and enhance maize molecular breeding. In a promising study of managing aflatoxin contamination in maize through host-induced RNAi-based gene silencing strategy (HIGS), Raruang et al. selected *A. flavus* gene *aflM* encoding versicolorin dehydrogenase, a key enzyme involved in the aflatoxin biosynthetic pathway, as a target for suppression through HIGS. They reported up to 76.4% reduction in aflatoxin levels in the transgenic lines containing the HIGS construct targeting the *aflM* in comparison to the null controls under field inoculation conditions. Likewise, they further indicated that genetic transformation to suppress the fungal target genes through HIGS can also protect grains from post-harvest aflatoxin contamination since they reported a 95.3% reduction in aflatoxin levels of harvested transgenic maize kernels compared to the null kernels during a 7-day incubation under 100% humidity. This enhanced aflatoxin resistance was correlated to the presence of high levels of *aflM*-specific small RNAs. Transferring the resistant trait from these transgenic lines into elite maize background resulted in a 60–80% reduction in aflatoxin of the F1 crosses under field conditions. This study offered a more sustainable approach in managing aflatoxin contamination in maize and other susceptible crops. In an effort to reduce aflatoxin accumulation in maize through identifying associated quantitative trait loci (QTL), Womack et al. provided the map of QTL by a bi-parental population comprised of 241 F2:3 families derived from the cross of inbred lines Mp705 (susceptible) × Mp719 (resistant) in maize. The mapping population was characterized in replicated field trials in three environments for resistance to aflatoxin accumulation under artificial inoculation with an *A. flavus* spore suspension. The genetic linkage map was constructed with 1,276 single nucleotide polymorphism (SNP) and simple sequence repeat (SSR) molecular markers covering a total genetic distance of 1,642 cM across all ten maize chromosomes. The authors concluded that the aflatoxin-reducing QTL in the chromosomal regions bin 1.06 and 3.09 are critically important for developing aflatoxin-resistant maize lines and hybrids and should be the primary targets to elite lines with marker-assisted breeding.

Biological control is another important approach to mitigate aflatoxin contamination of crops and agricultural commodities in pre- and post-harvest conditions. A promising approach is the use of competitive atoxigenic strains of *A. flavus* in the field. Over a 4-year period, Weaver and Abbas applied three biocontrol strains of *A. flavus* (NRRL 21882, 18543, and 30797) annually, to the 3.2-ha commercial corn field in the Mississippi Delta. They showed that after 4 years of biocontrol applications, the *A. flavus* population recovered from the grain was approximately 11% aflatoxigenic, regardless of the particular biocontrol treatment. As a conclusion, they indicated that biocontrol applications could be beneficial when the initial soil population has a high percentage of aflatoxigenic isolates. In a 10-year study as a field trial, Bandyopadhyay et al. applied Aflasafe, an advanced biocontrol-based product employing atoxigenic strains of *A. flavus* to control aflatoxin contamination of maize and groundnut in Nigeria. They showed that efficacy of the biocontrol product in limiting aflatoxin contamination was stable regardless of farming practices, crop varieties, or environmental challenges. Pertaining to this long-term efficient technology of aflatoxin management, the authors indicated that biocontrol could be considered as a preferred route for aflatoxin management. It also contributes to better health, increased income, and greater trading opportunities for groundnut and maize farmers throughout the world.

Climatic conditions of temperature and water activity together with drought stress are another important aspects of aflatoxin contamination of crops. In a study by Gasperini et al. post-harvest control of AFB_1_ by non-toxigenic strains of *A. flavus* in non-GM and isogenic GM maize cultivars was evaluated with special focus on environmental conditions affecting fungal growth and aflatoxin production. The authors concluded that pre-harvest ripening stage of maize cobs and their inherent water availability, interacting variables such as type of cultivar, *T*°C, CO_2_ levels, and water availability conditions, resilencing of the non-toxigenic strains and finally formulation approaches of applied biocontrol agents affect pre- and post-harvest fungal growth and subsequent aflatoxin contamination of maize in practice. Hanano et al. studied the role of Caleosin/Peroxygenase system in elevating the virulence of *A. flavus* through increasing sporulation and aflatoxigenicity after exposure of the fungus to an environmental pollutant toxin named “2,3,7,8-tetrachlorinated dibenzo-*p*-dioxin” (TCDD). The authors highlighted the impact of climate changes on food safety as new challenges in a global context and the necessity of reinforce the global regulations of food and feed products.

Another important challenge of aflatoxin control is the use of natural inhibitors of growth and aflatoxin production by the fungus. In an effort to control aflatoxin production by *A. flavus* in laboratory conditions by plant-derived natural molecules, Nobili et al. used non-digestible hull of common buckwheat, *Fagopyrum esculentum* extracted by supercritical fluid extraction process using carbon dioxide (SFE-CO_2_) to inhibit fungal growth and toxin production. They showed that SFE-CO2 extract of *F. esculentum* not only efficiently inhibited *A. flavus* growth and aflatoxin production *in vitro* probably due to containing higher amounts of polyphenols and lipophilic bioactive molecules but also it could be considered for open field applications considering its solvent-free nature which reduces the potential risk for plants and the environment. Gong et al. examined antifungal volatiles of a soil isolate of *Alcaligenes faecalis* to control *A. flavus* growth and aflatoxin production in groundnut, maize, rice and soybean during storage. They identified more than 25 compounds in the volatiles of *A. faecalis*, of which two compounds i.e., disulfide dimethyl (DMDS) and methyl isovalerate (MI) were proven to be responsible for antifungal properties and thus, they may be considered as promising agents in biocontrol of *A. flavus* in practice. In a comprehensive review on targeting alternative oxidase (AOX), a crucial fungal enzyme that affects fungal pathogenesis, morphogenesis, stress signaling, drug resistance and even mycotoxin production in the case of sterigmatocystin as a precursor of aflatoxin biosynthesis, Tian et al. highlighted that AOX inhibitors alone or in combination with other known antifungals should be considered as promising antifungals against *A. flavus* growth and aflatoxin contamination of crops. They indicated that further understanding of fungal alternative respiration and fungal AOX structure, and screening of effective fungal-specific AOX inhibitors are needed for practical application of AOX in food industries.

Detoxification of aflatoxins and rapid screening techniques of elimination of contaminated grains are important aspects of mycotoxin research due to the unfavorable fungal growth and subsequent aflatoxin contamination of food and feed in many tropical countries and economic problems of contaminated food elimination in low-income countries. In a promising effort in develop a rapid screening method and detoxify contaminated grains by using a new strategy, Juodeikiene et al. used an acoustic strategy in combination with fermentation by modeling maize and nuts contaminated with different amounts of aflatoxins. They successfully used a portable acoustic spectrometer comparable with ELISA in cost and speed for high-throughput detection of aflatoxins in grains and nuts to eliminate contaminated ones from the production chain and also they achieved detoxifying aflatoxin-contaminated grains through ethanol fermentation as a novel approach. In a non-destructive rapid image-based screening, Hruska et al. showed the usefulness of fluorescence hyperspectral imaging to differentiate susceptible and resistant corn hybrids infected by a toxigenic and atoxigenic strains of *A. flavus*. They indicated a significant role for the intensity of fluorescence when using fluorescence hyperspectral imaging for early detection of maize kernels infected with toxigenic and atoxigenic *A. flavus* in resistant and susceptible corn varieties.

Host-fungus interaction is another challenging area of aflatoxin research due to its dynamic nature. Musungu et al. used systems biology approach involving transcriptomic dual RNA-seq to uncover interactions between *A. flavus* and *Zea mays*. They showed that the activation of *Z. mays* resistance genes effectively influenced the expression of specific *A. flavus* genes. As a result, transcripts and pathways of *A. flavus* contributed to endosomal transport, aflatoxin production, and carbohydrate metabolism were up-regulated. Sweany and Damann studied the dual challenging effects of volatiles produced by *A. flavus* on aflatoxin production. They showed that these volatiles either stimulate aflatoxin production or suppress toxin production by the fungus *in vitro*. They concluded that the contribution of fungal volatiles to quorum sensing and communication. Applying them in modified atmospheres during grain storage with the aim of minimizing aflatoxin contamination warrants further investigation.

In a well-designed review by Arenas-Huertero et al. on the role of aryl hydrocarbon receptor (AhR) pathway in aflatoxins activation, it has been shown that the mono-oxygenases, such as CYP1A1 which are expressed abundantly in the liver as aflatoxin activators, have elemental promoters which are used by the AhR activation. With evidences of *in vitro* induction of an aflatoxin B_1_-induced increase in CYP1A activity and CYP1A transcription, in association with an enhanced AhR activity, the authors suggested that AhR pathway activation may be considered as a toxicity mechanism of AFB_1_.

Human exposure to aflatoxins is another challenging but not well-studied area. In the concise overview of aflatoxin contamination of foods and related biomarker research, Yunus et al. described aflatoxin contamination of milk as a public health problem in Pakistan and how these problems have influenced the Pakistan population by highlighting the aflatoxin concentrations in basic food products and their comparison with established aflatoxin limits. They showed that around 80% of AFM_1_ in milk was due to the use of contaminated cottonseed cake in dairy rations. The authors suggested a replacement of cottonseed cake with feedstuffs lower in aflatoxin contamination such as canola meal, and commercial concentrate feeds. They concluded that long-term mitigation strategies could be successfully used to reduce aflatoxin contamination in cottonseed cake and increase the safety of dairy animal feed. In a study by Ismail et al. on aflatoxin contamination of commercially available black tea in Pakistan, the authors estimated daily intake (EDI) of aflatoxins through branded and non-branded black tea consumption and the health risk assessment based on the margin of exposure (MOE) approach. As a result, they showed that aflatoxin contamination was common in both types of tea samples and the MOE values for aflatoxins after black tea consumption indicated a considerable public health risk problem. Results indicated the possible transfer of more than half of aflatoxins from tea leaves to the tea beverage and probable production of aflatoxins degradation products probably due to four to five times boiling of tea. The authors suggested that level of aflatoxins should be monitored constantly in tea and tea products by the health and regulatory agencies.

In conclusion, this Research Topic opens exciting perspectives on challenges and opportunities in mitigating aflatoxins in food and agricultural crops and their global health impacts with a special focus on the development of suitable strategies for preventing toxigenic fungal growth in the field and storage, thereby reducing or eliminating subsequent aflatoxin contamination of the food and feed supplies.

## Author Contributions

MR-A, Z-YC, MS-G, and MR designed the study. MR-A and MS-G prepared the draft. All authors read and approved final version of the manuscript. MR-A supervised the study. All authors contributed to the article and approved the submitted version.

## Funding

This work was funded by the Pasteur Institute of Iran and by Elite Researcher Grant Committee under award numbers 958634 and 963646 from the National Institute for Medical Research Development (NIMAD) to MR-A are appreciated.

## Conflict of Interest

The authors declare that the research was conducted in the absence of any commercial or financial relationships that could be construed as a potential conflict of interest.

## Publisher's Note

All claims expressed in this article are solely those of the authors and do not necessarily represent those of their affiliated organizations, or those of the publisher, the editors and the reviewers. Any product that may be evaluated in this article, or claim that may be made by its manufacturer, is not guaranteed or endorsed by the publisher.
